# Global health status and fatigue score in isocitrate dehydrogenase-mutant diffuse glioma grades 2 and 3: A longitudinal population-based study from surgery to 12-month follow-up

**DOI:** 10.1093/nop/npae017

**Published:** 2024-03-01

**Authors:** Tomás Gómez Vecchio, Isabelle Rydén, Anneli Ozanne, Malin Blomstrand, Louise Carstam, Anja Smits, Asgeir Store Jakola

**Affiliations:** Department of Clinical Neuroscience, Institute of Neuroscience and Physiology, Sahlgrenska Academy, Gothenburg University, Gothenburg, Sweden; Department of Clinical Neuroscience, Institute of Neuroscience and Physiology, Sahlgrenska Academy, Gothenburg University, Gothenburg, Sweden; Department of Neurology, Sahlgrenska University Hospital, Gothenburg, Sweden; Department of Neurology, Sahlgrenska University Hospital, Gothenburg, Sweden; Institute of Health and Care Sciences, Sahlgrenska Academy, Gothenburg University, Gothenburg, Sweden; Department of Oncology, Institute of Clinical Sciences, Sahlgrenska Academy, Gothenburg University, Gothenburg, Sweden; Department of Oncology, Sahlgrenska University Hospital, Gothenburg, Sweden; Department of Clinical Neuroscience, Institute of Neuroscience and Physiology, Sahlgrenska Academy, Gothenburg University, Gothenburg, Sweden; Department of Neurosurgery, Sahlgrenska University Hospital, Gothenburg, Sweden; Department of Clinical Neuroscience, Institute of Neuroscience and Physiology, Sahlgrenska Academy, Gothenburg University, Gothenburg, Sweden; Department of Neurology, Sahlgrenska University Hospital, Gothenburg, Sweden; Department of Clinical Neuroscience, Institute of Neuroscience and Physiology, Sahlgrenska Academy, Gothenburg University, Gothenburg, Sweden; Department of Neurosurgery, Sahlgrenska University Hospital, Gothenburg, Sweden

**Keywords:** fatigue, glioma, grade 2, grade 3, HRQoL

## Abstract

**Background:**

At the group level, health-related quality of life (HRQoL) in patients with IDH-mutant diffuse glioma grades 2 and 3 seems to remain stable over time. However, clinical experience indicates that there are patients with unfavorable outcomes on key HRQoL subdomains. The aim of this longitudinal population-based study, following patients over a period of 12 months from surgery, was to describe individual-level data on global health status and fatigue score and explore possible predictors of deterioration.

**Methods:**

All patients undergoing surgery for presumed glioma grades 2 or 3 at the Sahlgrenska University Hospital during 2017–2022, were screened for the study. Patients were invited to complete the European Organization of Research and Treatment of Cancer core questionnaires and brain module at baseline, 3 and 12 months postoperatively. Data is reported with respect to minimal clinical important difference (MCID).

**Results:**

We included 51 patients with IDH-mutant diffuse glioma grades 2 or 3. There was no difference in group-level data of either global health status or fatigue score from baseline to the 12-month follow-up (*P*-value > .05). Unfavorable individual changes (beyond MCID) in global health status and fatigue score were observed in 12 and in 17 patients, respectively (23.5% and 33.3%). A lower proportion of proton radiotherapy was found in patients with unfavorable changes in fatigue (10/15, 66.7%) compared to all other patients undergoing radiotherapy (22/23, 95.7%, *P*-value .03).

**Conclusions:**

Deterioration beyond MCID was seen in approximately one-third of patients. Changes in global health status could not be predicted, but changes in fatigue may be influenced by tumor-targeted and symptomatic treatment.

Patients with isocitrate dehydrogenase (IDH) mutant diffuse glioma grades 2 and 3 show a variety of symptoms. Symptoms may be caused by the tumor itself, by tumor-targeting and symptomatic treatment, or by a combination of these.^1^ Common symptoms include neurological and cognitive deficits, mood disorders, fatigue, epileptic seizures, and sometimes signs of intracranial pressure.^1–3^ Symptom burden in patients with glioma seems to be mostly related to the symptoms of a brain disease rather than to symptoms often associated with cancer patients.^4^ There is evidence to sustain that symptoms and health-related quality of life (HRQoL) are highly interrelated in patients with glioma.^5^ Consequently, various interacting factors can negatively impact the patient’s HRQoL.

Issues concerning fatigue in this patient group have gained increased attention in recent years.^6,7^ Fatigue is a multidimensional concept expressing physical, cognitive, and affective concerns. It is known to be a prominent symptom following low-grade glioma treatment.^8,9^ However, clinical experience shows us that it is difficult to predict long-term outcomes on self-reported HRQoL, particularly on constructs such as fatigue and global health status. Furthermore, there seems to be a heterogeneity in patients’ trajectories, showing contrasting results between group and individual level analysis.^10,11^

Studies in patients with mixed glioma cohorts, including low-grade gliomas, reporting on group-level data utilize diverse instruments including generic assessments of HRQoL.^12,13^ The most frequently used instruments to estimate HRQoL after glioma surgery are the European Organization of Research and Treatment of Cancer (EORTC) core questionnaires (QLQ-C30) and brain modules (QLQ-BN20).^12–14^ More studies focusing on HRQoL in patients with low-grade glioma employ a cross-sectional rather than a longitudinal design, and those employing a longitudinal design often use non-standardized timepoints.^13^ Thus, we believe that longitudinal changes of self-reported HRQoL have not been consistently reported in cohorts consisting exclusively of patients with IDH-mutant diffuse glioma grades 2 and 3. Compared to patients with glioblastoma, these patients have a favorable prognosis with a median survival time exceeding 10 years.^15^ This prompted us to study longitudinal individual-level data to better capture the landscape of their individual trajectories.

In this longitudinal population-based study, following patients over a period of 12 months from surgery, we aim to describe individual-level data in patients with IDH-mutant diffuse glioma grades 2 and 3 using the constructs global health status and fatigue score from the EORTC QLQ-C30 and QLQ-BN20 HRQoL questionnaires. In addition, we explore possible predictors of deterioration.

## Material and Methods

### Inclusion Criteria

All adult patients undergoing surgery at the Neurosurgical Department at Sahlgrenska University Hospital, Sweden, from February 2017 to February 2022 with an initial radiological diagnosis indicating a glioma grades 2 or 3 were consecutively invited to participate in the study. Patients were prospectively recruited and followed up 1 year after surgery. Postoperatively, all tumors were classified according to the 2021 World Health Organization Classification of Tumors of the Central Nervous System.^16^ Patients with a diagnosis other than IDH-mutant diffuse glioma grades 2 or 3, or patients undergoing reoperation within the 12-month follow-up were excluded.

The study was conducted in accordance with the Declaration of Helsinki, the study protocol was approved by the Regional Ethical Review Board in Gothenburg, Sweden (Dnr: 1067-16). As all cases are discussed at weekly multidisciplinary meetings, all patients presenting with supratentorial-located presumed intra-axial tumors without significant contrast enhancement were approached. All patients included in the study provided written informed consent.

### Outcomes and Follow-up

At baseline (ie, prior to surgery, usually within a week after the initial neurosurgical outpatient consultation) and at 3 and 12 months postoperatively, all patients were invited to complete self-reported HRQoL questionnaires in conjunction with a routine clinical visit. Disease-specific HRQoL was assessed using 2 instruments elaborated by EORTC: QLQ-C30 and QLQ-BN20.^17,18^ Good validity of QLQ-C30 was recently shown in patients with localized to advanced cancer and QLQ-BN20 was validated in 2010, demonstrating adequate psychometric properties.^19,20^ Together, the QLQ-C30 and QLQ-BN20 include 50 questions from which 26 scales and items were scored. The scales and items scores range from 0 to 100. For status and functioning, a high score represents a healthier level of functioning; for dysfunctions/deficits/symptoms, a high score represents a higher level of symptomatology.

Clinical variables for this study were retrospectively extracted from electronic medical records. Details on antitumor and symptomatic treatment were retrospectively assessed in all patients. Neurological deficits (motor, language, cognitive, and visual), new-onset seizures, and seizure control were routinely assessed at admission, discharge, and 3-month follow-up. Worsening of seizure control was defined as increased severity (eg, from focal preoperatively to general postoperatively) and/or increased frequency. Both clinical scenarios are typically followed by adjustment of treatment. Data on neurological deficits and seizures were also retrospectively collected from the electronic medical records reflecting their status at baseline and at the 3-month follow-up. The first follow-up was performed at 3 months since the 3-month time-point for evaluation of deficit after surgery is often used for the distinction of transient and permanent deficits related to the surgical treatment.^21,22^ Neurological deficit was considered permanent if present at 3 months postoperatively, either as new-onset or deteriorated compared to baseline.

### Statistics

Dropout rate was calculated separately at each follow-up by dividing the number of participants who did not attend a follow-up by the total number of participants assessed at baseline. Datapoints missing completely at random were imputed by the items’ mean of the observed values.^23^ In addition to global health status and fatigue score, individual changes in QLQ-C30 and QLQ-BN20 were calculated for selected scales and items (global health status, physical functioning, cognitive functioning, motor dysfunction (MD), vision disorder (VD), communication deficit (CD), seizures, and fatigue), ie, those considered of special interest to neurosurgeons due to their relationship with traditional neurologic deficit. The selection of scales and items included in the analysis of individual change was done prior to data analysis, choosing the scales and items that reflected the neurological deficits reported in this study.

Individual changes were calculated by subtracting follow-up values from baseline values. Assessment of minimal clinically important difference (MCID) was based on available literature.^11,24^ A value of 10 points for all QLQ-C30 and QLQ-BN20 scales and items was used as MCID to group patients in this study.^25–28^ In a sensitivity analysis on global health status and fatigue score, anchor-based values for MCID were used from the literature (6 and 8 points, respectively).^29^

Statistical analysis was performed using IBM SPSS Statistics 28 (IBM Corp., Armok, NY, USA). Central tendencies for descriptive statistics are presented either with percentages, means with standard deviation (SD), or medians with first and third quartiles (Q1, Q3). Independent *t*-test or Fisher-Freeman-Halton exact test were used to analyze differences between groups. Distribution-based interpretations were supported with pair sample *t*-test and Cohen’s effect size.

Spearman’s rank order correlations and Spearman rank order partial correlations were used for variable selection including all variables from Table 1. Partial correlations are presented in Supplementary Materials with Spearman correlation coefficient (r) and *P*-values. Partial correlations include 95% confidence intervals (CI). Bonferroni correction was applied adjusting the *P*-value to the total number of variables included in the analysis. For all correlations, the significance level was set to < 0.001.

**Table 1.** Patient Characteristics, Treatments, and Clinical Outcomes in Patients Completing the 12-Month Follow-up (*N* = 51)

Variable	Study Sample
Age at surgery, mean (SD)	43 (11.9)
Female, No (%)	26 (51)
KPS at admission, median (Q1, Q3)	90 (80–90)
Incidental, No (%) History of seizures at admission, No (%)	8 (15.7) 34 (66.7)
Neurological deficit at admission, No (%)	
Motor	3 (5.9)
Cognitive	6 (11.8)
Visual	3 (5.9)
Language	5 (9.8)
Any neurological deficit at admission, No (%)	15 (29.4)
Tumor location, No (%)	
Mainly frontal	29 (56.9)
Mainly temporal	9 (17.6)
Other	13 (25.5)
Choice of neurosurgical intervention, No (%)	
Tumor resection	48 (94.1)
WHO 2021 classification, No (%)	
Oligodendroglioma, grade 2	14 (27.5)
Astrocytoma, grade 2	19 (37.3)
Oligodendroglioma, grade 3	12 (23.5)
Astrocytoma, grade 3	6 (11.7)
Treatment before 12-month follow-up, No (%)	
None	8 (15.7)
Chemotherapy only^a^	5 (9.8)
Radiochemotherapy^a^^,^^b^	38 (74.5)
Radiation type, proton	32 (62.7)
Radiation type, photon	6 (11.8)
Other relevant treatments and interventions, No (%)	
Antiepileptic drug use at baseline	34 (66.7)
Antiepileptic drug use at 12-month follow-up^*^	33 (64.7)
Physical rehabilitation^*^	9 (17.6)
Postoperative seizures^c^, No (%)	5 (9.8)
New or worsened neurological deficit^d^, No (%)	
Motor	11 (21.6)
Cognitive	7 (13.7)
Visual	2 (3.9)
Language	11 (21.6)
Any new or worsened neurological deficit, No (%)	22 (43.1)
Any permanent, new or worsened, neurological deficit, No (%)	12 (23.5)

^a^Either temozolomide, lomustine or procarbazine hydrochloride, lomustine and vincristine (PCV).

^b^Either concomitant or adjuvant.

^c^From surgery up to the 3-month follow-up. New or worsened.

^d^From surgery up to the 3-month follow-up. Transient or permanent.

^*^Observe that 5 patients are missing data on AED use at 12 months and 1 patient is missing data on physical rehabilitation.

Simple linear regressions were performed with change in global health status at 12-month follow-up and change in fatigue score at the 12-month follow-up as response variables. Based on the correlation analysis, the variables age at surgery, sex, Karnofsky performance status scale at admission, history of seizures at admission, choice of neurosurgical intervention, tumor type, tumor grade, postoperative treatment before the 12-month follow-up, antiepileptic drug (AED) use at the 12-month follow-up, and any permanent new or worsened neurological deficits up to the 3-month follow-up were used as predictor variables. All statistical tests were 2-tailed with a significance level of < 0.05 if not otherwise specified.

## Results

### Attrition Rate and Missing Data

Of the initial 118 patients screened for the study, 51 had a diagnosis other than IDH-mutant diffuse glioma grades 2 or 3 and were excluded from the study. No patient underwent reoperation due to progressive disease within the 12-month follow-up. The participation rate at baseline was 83.6% (56/67 patients). For the 11 excluded patients diagnosed with IDH-mutant diffuse glioma grades 2 or 3, there were missing baseline data due to acute surgery (6 patients), administrative problems (4 patients), or declined participation (1 patient).

Of the included 56 patients, 50 responded at the 3-month follow-up and another subgroup of 51 (of the initial 56 patients) responded at the 12-month follow-up. Hence, the dropout rate was 11% at 3 months and 9% at the 12-month follow-up. The mean time to follow-up was 3.5 (SD 0.9) and 12.6 (SD 2.0) months, respectively. Additionally, patients missing their appointments at the 3-month (6 patients) and the 12-month follow-up (5 patients) did not seem to diverge (in terms of demographic characteristics) from the rest of the cohort. Four patients missed both the 3-month and the 12-month follow-up. Of the 5 patients missing the 12-month follow-up, 2 declined participation, 2 missed the appointment due to worsening health status, and 1 died before the follow-up due to causes unrelated to the primary tumor diagnosis or its treatment.

A total of 157 questionnaires were completed. Of the 7850 data points included in the 157 questionnaires, only 8 were missing completely at random (4 data points at baseline, 3 data points at the 3-month follow-up, and 1 data point at the 12-month follow-up). These 8 missing values were imputed by the items' mean of the observed values. The final cohort for the 12-month longitudinal study included 51 patients.

### Patient Characteristics, Treatments, and Clinical Outcomes at Baseline

Of the 51 patients completing the 12-month follow-up, the mean age was 43 years, and 26 (51%) were females. At admission, the median Karnofsky performance status was 90. While 8 (15.7%) of the tumors were incidentally discovered, 34 (66.7%) of the patients had a history of seizures, and 15 (29.4%) showed signs of neurological deficit at admission. The most common choice of surgical intervention was tumor resection (48 patients, 94.1%). Twenty-six (51%) and 25 (49%) patients were diagnosed with oligodendroglioma and astrocytoma, respectively, and tumors were graded as WHO grade 2 in 33 (64.7%) cases. Before the 12-month follow-up, 38 (74.5%) patients underwent radiotherapy, either proton (32 patients) or photon therapy (6 patients). Baseline patient characteristics, treatments, and clinical outcomes are presented in Table 1. Additionally, baseline patient characteristics, treatments, and outcomes for all patients included at baseline by tumor type and at group level (including 3-month and 12-month follow-ups) for all scales and items of the QLQ-C30 and QLQ-BN20 are presented in Supplementary Materials.

### Individual Change Beyond MCID in QLQ-C30 and QLQ-BN20 Selected Scales and Items at 12-month Follow-up

There were 22 patients (43.1%) reporting improvement beyond MCID in global health status while 12 patients (23.5%) reported a decline beyond MCID. The proportion of patients reporting decline beyond MCID in cognitive functioning was 15/51 (29.4%) while 18/51 (35.3%) reported improvement beyond MCID. The corresponding proportions for changes in physical function were 14/51 (27.5%) and 5/51 (9.8%), for a decline beyond MCID and improvement beyond MCID, respectively.

**Figure 1.**
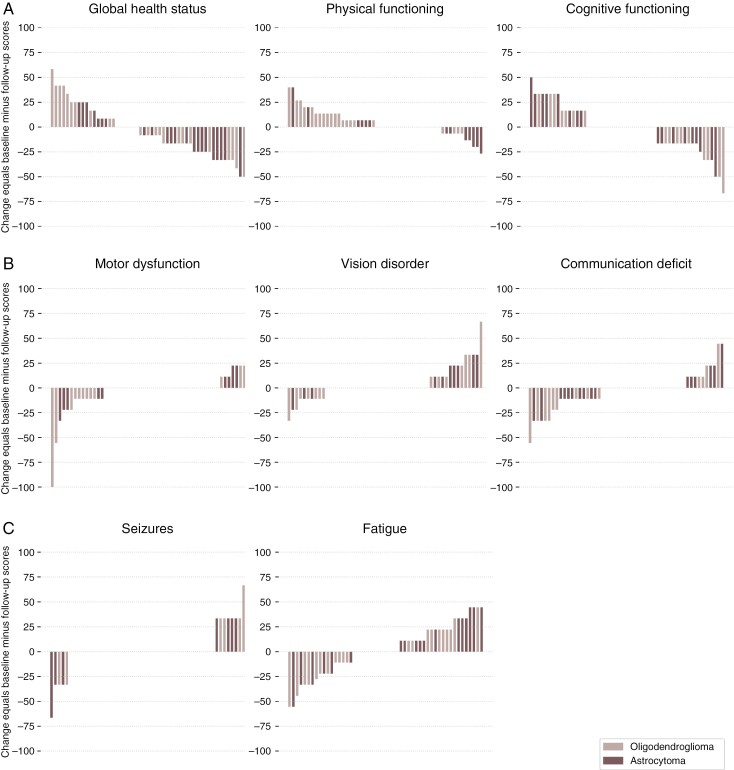
(A) Status and functioning scores, (B) Dysfunction and Deficits and (C) Symptoms. The bar plots show the individual change in the selected scales and items of the European Organization of Research and Treatment of Cancer QLQ-C30 and QLQ-BN20. Change was calculated subtracting the 12-month follow-up scores from baseline scores. Every bar represents a single individual (*N* = 51). Color labels indicate tumor type. *Note: in status and functioning scores negative values indicate improvement; in dysfunctions, deficits and symptom scores positive values indicate improvement.*

Concerning MD, VD, CD, and seizures (S) scores, the most common response was a non-MCID change in symptom burden (MD 30/51 [58.8%], VD 27/51 [52.9%], CD 22/51 [43.1%], and S 38/51 [74.5%] patients). The proportion of patients reporting improvement beyond MCID in fatigue (22/51, 43.1%) was higher than the proportion reporting deterioration beyond MCID (17/51, 33.3%). The scores are summarized on individual level in Figure 1.

A comparison of changes within the selected QLQ-C30 and QLQ-BN20 scales/items between patients with oligodendroglioma or astrocytoma demonstrated that patients with oligodendroglioma reported a statistically significant decline in physical functioning compared to patients with astrocytoma (mean 9.0 [SD 11.6], and −0.8 [SD 13.1], respectively, *P*-value .01). Additional comparisons are presented in Supplementary Materials.

### Risk Factors of Change in Global Health Status and Fatigue Score at 12-Month Follow-up

As seen in the MCID-based analyses and Figure 1, global health status and fatigue scores were the QLQ-C30 and QLQ-BN20 scales/items that were most often subject to any change at 12-month follow-up (i.e., not consisting of a large group without any change). These 2 variables were therefore considered more likely to be informative in analyses of predictors of change and were selected for further analyses.

Patient characteristics and treatment details in 2 groups of selected patients (12 patients with an unfavorable change beyond MCID in global health status and 17 patients with an unfavorable change beyond MCID in fatigue score) were compared to all other patients. Additional risk factors included in the analyses were history of seizures, tumor location, and details of the postoperative treatment.

For patients with an unfavorable change beyond MCID in global health status, none of the chosen predictors were significantly differently distributed compared to all other patients (Table 2). For the comparison of patients grouped by an unfavorable change beyond MCID in the fatigue score, the only statistically significant difference between groups was a lower proportion of proton radiotherapy in patients with an unfavorable change beyond MCID in the fatigue score (66.7%) compared to all other patients undergoing radiotherapy (95.7%, *P*-value .03; Table 3).

**Table 2.** Patients With an Unfavorable Change Beyond Minimal Clinical Important Difference in Global Health Status at the 12-Month Follow-up Compared to All Other Patients (*N* = 51)

Variable	Unfavorable Change Beyond MCID in Global Health Status at 12-Month Follow-up (*N* = 12)	All Other Patients (*N* = 39)	*P*-value
Age at surgery, mean (SD)	48.1 (9.8)	41.5 (12.2)	.07
Female, No (%)	5 (41.7)	21 (53.8)	.52
KPS < 80 at admission, No (%)	1 (8.3)	5 (12.8)	1.0
Incidental, No (%)	2 (16.7)	6 (15.4)	1.0
History of seizures at admission, No (%)	8 (66.7)	26 (66.7)	1.0
Any neurological deficit at admission, No (%)	5 (41.7)	10 (25.6)	.30
History of seizures at admission without any neurological deficit, No (%)	5 (41.7)	20 (51.3)	.74
Tumor location, No (%)			
Mainly frontal	7 (58.3)	22 (56.4)	1.0
Mainly temporal	1 (8.3)	8 (20.5)	.67
Other	4 (33.3)	9 (23.1)	.47
Tumor laterality, mainly left hemisphere, No (%)	3 (25.0)	20 (51.3)	.18
Choice of neurosurgical intervention, No (%)			
Tumor resection	12 (100)	36 (92.3)	1.0
Tumor type, oligodendroglioma, No (%)	8 (66.7)	18 (46.2)	.32
Tumor grade, grade 2, No (%)	8 (66.7)	25 (64.1)	1.0
Treatment before 12-month follow-up			
Start chemotherapy (delta T in months), mean (SD)	4.5 (2.1)	4.4 (2.4)	.93
Start radiotherapy (delta T in months), mean (SD)	3.8 (2.4)	3.4 (2.6)	.68
None, No (%)	3 (25.0)	5 (12.8)	.37
Chemotherapy only^a^, No (%)	0 (0)	5 (12.8)	.32
Radiochemotherapy^a^^,^^b^, No (%)	9 (75.0)	29 (74.4)	1.0
Other treatment details			
Choice of chemotherapy first line drugs, temozolomide, No/*N* (%)	4/9 (44.4)	21/34 (61.8)	.46
Radiation type, proton, No/*N* (%)	7/9 (77.8)	25/29 (86.2)	.61
Radiation mean dose to the brain, GyRBE^c^, mean (SD)	14.4 (5.0)	14.1 (5.0)	.85
Other relevant treatments and interventions, No/*N* (%)			
Antiepileptic drug use at 12-month follow-up^*^	10/11 (90.9)	23/35 (65.7)	.14
Physical rehabilitation^*^	4/11 (36.4)	5/39 (12.8)	.09
Any permanent, new or worsened, neurological deficit, No (%)	5 (41.7)	7 (17.9)	.12

^a^Either temozolomide, lomustine, or procarbazine hydrochloride, lomustine and vincristine (PCV).

^b^Either concomitant or adjuvant.

^c^Biologic equivalent dose with fractions of 2Gy (α/β3).

^*^Observe missing data.

**Table 3.** Patients With an Unfavorable Change Beyond Minimal Clinical Important Difference in the Fatigue Score at the 12-Month Follow-up Compared to All Other Patients (*N* = 51)

Variable	Unfavorable Change Beyond MCID in Fatigue Score at 12-Month Follow-up (*N* = 17)	All Other Patients (*N* = 34)	*P*-value
Age at surgery, mean (SD)	45.8 (11.5)	41.5 (12.1)	.25
Female, No (%)	6 (35.3)	20 (58.8)	.14
KPS < 80 at admission, No (%)	1 (5.9)	5 (14.7)	.65
Incidental, No (%)	2 (11.8)	6 (17.6)	.70
History of seizures at admission, No (%)	12 (70.6)	22 (64.7)	.76
Any neurological deficit at admission, No (%)	5 (29.4)	10 (29.4)	1.0
History of seizures at admission without any neurological deficit, No (%)	10 (58.8)	15 (44.1)	.38
Tumor location, No (%)			
Mainly frontal	11 (64.7)	18 (52.9)	.55
Mainly temporal	3 (17.6)	6 (17.6)	1.0
Other	3 (17.6)	10 (29.4)	.50
Tumor laterality, mainly left hemisphere, No (%)	7 (41.2)	16 (47.1)	.77
Choice of neurosurgical intervention, No (%)			
Tumor resection	16 (94.1)	32 (94.1)	1.0
Tumor type, oligodendroglioma, No (%)	11 (64.7)	15 (44.1)	.24
Tumor grade, grade 2, No (%)	9 (52.9)	24 (70.6)	.23
Treatment before 12-month follow-up			
Start chemotherapy (delta T in months), mean (SD)	5.1 (2.6)	4.0 (2.1)	.18
Start radiotherapy (delta T in months), mean (SD)	3.7 (2.6)	3.4 (2.5)	.72
None, No (%)	2 (11.8)	6 (17.6)	.70
Chemotherapy only^a^, No (%)	0 (0)	5 (14.7)	.16
Radiochemotherapy^a^^,^^b^, No (%)	15 (88.2)	23 (67.6)	.18
Other treatment details			
Choice of chemotherapy first line drugs, temozolomide, No/*N* (%)	6/15 (40.0)	19/28 (67.9)	.11
Radiation type, proton, No/*N* (%)	10/15 (66.7)	22/23 (95.7)	.03 ^(^^*^^)^
Radiation mean dose to the brain, GyRBE^c^, mean (SD)	14.9 (5.7)	13.6 (4.5)	.48
Other relevant treatments and interventions, No/N (%)			
Antiepileptic drug use at 12-month follow-up^*^	12/15 (80.0)	21/31 (67.7)	0.50
Physical rehabilitation^*^	3/16 (18.8)	6/34 (17.6)	1.0
Any permanent, new or worsened, neurological deficit, No (%)	4 (23.5)	8 (23.5)	1.0

^a^Either temozolomide, lomustine or procarbazine hydrochloride, lomustine and vincristine (PCV).

^b^Either concomitant or adjuvant.

^c^Biologic equivalent dose with fractions of 2Gy (α/β3).

^*^Observe missing data.

^(*)^Observe that only 6 patients received photon-therapy compared to 32 receiving proton therapy.

In a sensitivity analysis, when anchor-based values for MCID were applied, there was an increase from 12 to 17 in the number of patients experiencing unfavorable change in global health status; for fatigue score, there was no difference in the number of patients experiencing unfavorable change. For the new group of patients with unfavorable change beyond anchor-based MCID on global health status, none of the chosen predictors were significantly differently distributed compared to all other patients.

### Linear Regression Analysis of Individual Change in Global Health Status and Fatigue Score at 12-Month Follow-up

In the linear regression analyses, we sought to identify predictors of individual change in global health status and fatigue score at 12-month follow-up. None of the predictor variables were found to be independent predictors of global health status. Concerning fatigue score, the “AED use at 12-month follow-up” was an independent predictor of unfavorable change in fatigue score on an individual level whereas “chemotherapy only” as postoperative treatment was independently associated with improved individual level fatigue score at 12-month follow-up (Table 4).

**Table 4.** Univariable Linear Regression (*N* = 51)

	Simple Linear Regressions Response Variable:			
	Individual Change in Global Health Status at 12-Month Follow-up^a^		Individual Change in Fatigue Score at 12-Month Follow-up^b^	
Predictor Variables	B 95% CI	*P*-value	B 95% CI	*P*-value
Age at surgery	−0.3, 0.9	.27	−1,1, 0.1	.10
Sex, female	−20.6, 7.9	.37	−11.7, 17.2	.70
KPS < 80 at admission	−32.6, 11.5	.34	−7.1, 37.0	.18
History of seizures at admission	−14.0, 16,4	.87	−21.6, 8.9	.41
Choice of neurosurgical intervention, resection	−17.4, 43.1	.85	−40.7, 20.6	.51
Tumor type, oligodendroglioma	−3.4, 24.7	.13	−21.6, 7.1	.31
Tumor grade, grade 2	−7.6, 22.1	.33	−4.7, 25.0	.18
No postoperative treatment before 12-month follow-up	−1.3, 36.8	.07	−16.9, 22.1	.76
Chemotherapy only before 12-month follow-up,	−37.2, 10.4	.26	2.0, 48.6	.03
Radiochemotherapy before 12-month follow-up,	−22.5, 10.2	.46	−30.0, 2.3	.09
Antiepileptic drug use at 12-month follow-up	−5.9, 27.4	.20	−35.3, −2.5	.03
Any permanent, new or worsened, neurological deficit up to 3-month follow-up	−0.3, 32.3	.05	−21.0, 13.1	.64

^a^In individual change in global health status negative values indicate improvement.

^b^In individual change in fatigue score positive values indicate improvement.

### Relation Between Individual Change in Global Health Status and Individual Change in Fatigue Score at 12-Month Follow-up

**Figure 2.**
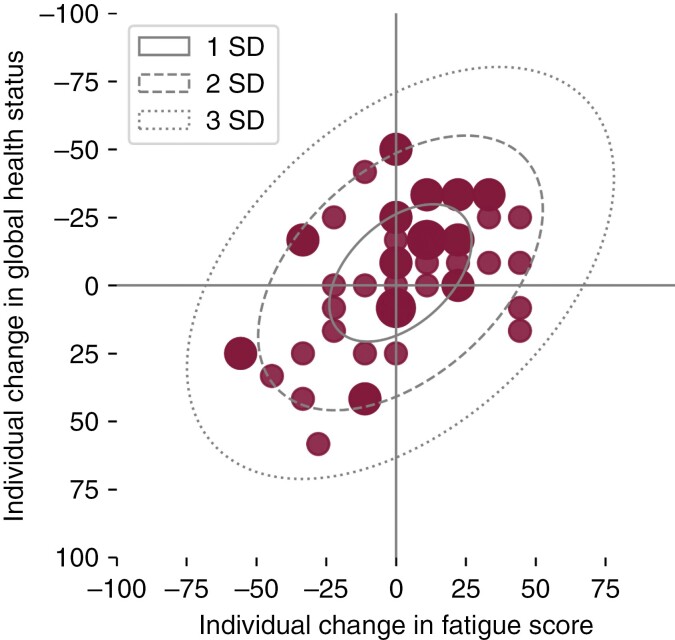
Scatter plot including confidence ellipse of covariance between individual change in global health status and individual change in fatigue score at the 12-month follow-up. Standard deviation calculated based on Pearson correlation coefficient. Small dots symbolize 1 patient, medium dots symbolize 2 patients, large dots symbolize 3 patients (*N* = 51). *Note:bottom-left indicates deterioration, upper-right indicates improvement.*

A scatter plot showing individual changes in global health status and individual changes in fatigue score highlighting the covariance at the 12-month follow-up is presented in Figure 2.

### Distribution-Based Interpretation at Group Level of Global Health Status and Fatigue Score Between Baseline and 12-Month Follow-up

There was no significant group-level difference in global health status over time (*P*-value .20, −4.58 [CI: −11.7 to 2.5], Cohen´s *d* −0.18 [CI: −0.46 to 0.10]). Mean global health status at baseline and at 12-month follow-up were 57.0 and 61.6, respectively. Neither was there any significant group-level difference in fatigue score over time (*P*-value .65, 1.63 [CI: −5.54 to 8.80], Cohen´s *d* 0.06 [CI −0.21 to 0.34]). Mean fatigue scores at baseline and at 12-month follow-up were 38.3 and 36.7, respectively. Additional results including baseline, 3-month and 12-month data are presented in Supplementary Materials.

## Discussion

In this longitudinal population-based study using self-reported HRQoL questionnaires in 51 patients with IDH-mutant diffuse glioma grades 2 and 3, a subgroup of patients reported unfavorable change beyond MCID in global health status (*n* = 12) and fatigue score (*n* = 17). At group level, however, there were no significant changes in either global health status or fatigue scores. A lower proportion of patients undergoing proton radiotherapy was found in patients with unfavorable change beyond MCID in the fatigue score when compared to all other patients undergoing radiotherapy (10 out of 15 patients, and 22 out of 23 patients, respectively). No significant predictors of individual change in global health status could be identified in our study. The parameters “use of AED at 12-month follow-up” and “chemotherapy only” were identified as predictors of individual change in the fatigue score.

Unlike other studies focusing on self-reported HRQoL, we set out to predict individual changes in global health status and fatigue score during the time span from admission to the neurosurgical department up to the 12-month follow-up visit. So far, most studies have aimed to assess the outcome of specific postoperative treatments. There are interesting similarities with our study. Due to the nature of their design, studies reporting changes in self-reported HRQoL from surgery to any follow-up tend to present data on mixed glioma groups.^30–34^ To the best of our knowledge there is to date only one published study that measured the effect of surgery on self-reported HRQoL in a cohort consisting exclusively of patients with IDH-mutant diffuse glioma grades 2 or 3.^35^ Altogether, regardless of study aims and time-point, self-reported HRQoL at group level seems to be stable over time in patients with glioma grades 2 or 3, including also previous WHO classifications.^36–45^ This was a finding in our study as well, where patients with negative symptom trajectories were balanced by those reporting symptom improvement. However, there is a knowledge gap concerning the possible causes of individual change in global health status and fatigue score in patients with IDH-mutant diffuse glioma grades 2 and 3. In this respect, it is interesting to note that none of the established prognostic factors for survival in this patient group, such as histological tumor type, patient age, and tumor location, could predict changes in global health status or fatigue in our cohort. This implies that, at least during the first year after surgery, there might be other—so far unknown—predictors of HRQoL, which are not directly related to the biological behavior of the tumor.

We focused on aspects of QLQ-C30 and QLQ-BN20 believed to be linked more directly to neurological and cognitive function. Although we had an intention to capture the impact of the surgical treatment at 3 months, the varying starting times of chemotherapy and radiotherapy made it difficult to interpret the data around this time point. An in-depth analysis (eg, including individually adapted timepoints for HRQoL assessment, and the use of a specific instrument to measure fatigue) would be preferable to explore the role of the independent predictors of individual change in fatigue score found in our study.

The cohort demographics together with aspects of HRQoL are comparable to what is observed in previous studies.^32,35^ These similarities support the external validity of the present study. There are, however, some limitations with our study related to the nature of the instrument (ie, ceiling and floor effects together with the characteristics of health and fatigue constructs) and the use of medical records to recollect clinical data. Although statistically significant, the presence of missing data made it difficult to draw a conclusion regarding the association of AED at the 12-month follow-up and individual change in fatigue score. Also, in the discussion we focused on studies with similar study designs and avoided those with cross-sectional designs and reports where patients with IDH-mutant diffuse glioma grades 2 or 3 were underrepresented. Acknowledging these limitations, together with the small sample size, we believe that the analysis presented here might once more raise awareness of the importance of analyzing individual-level data to provide a better overview of the HRQoL trajectories.

Despite our inability to find strong predictors, we found that one-quarter of our cohort suffered a deterioration in global health status beyond MCID. Similarly, one-third of our patients expressed an unfavorable change beyond MCID in their fatigue score from baseline to the 12-month follow-up. However, the numbers are small, and the associations found with fatigue could be due to selection bias in choice of postoperative therapy. We believe that our results might support qualitative studies, leading to a deeper understanding of patients’ symptom burden. Correspondingly, in patients with IDH-mutant diffuse glioma grades 2 and 3, there is also a need for interventional studies aiming to address the burden of the disease even years after primary treatment.

## Conclusion

Changes in global health status during the first year after surgery could not be predicted using routine clinical variables. However, changes in fatigue scores, observed during the same period, seemed to be associated with the combined effects of antitumor and symptomatic treatment. Further research is needed to assess the impact these treatments exert in patients with IDH-mutant diffuse glioma grades 2 and 3. By this, focus should be given to identifying patients at risk of developing unfavorable outcomes in global health status and fatigue score and providing relevant interventions.

## Supplementary Material

npae017_suppl_Supplementary_Material
