# Supplementary material for: Global health status and fatigue score in isocitrate dehydrogenase-mutant diffuse glioma grades 2 and 3: A longitudinal population-based study from surgery to 12-month follow-up
Source: Neurooncol Pract. 2024 Mar 1;11(3):347–57. doi: 10.1093/nop/npae017 (PMC11085849; doi:10.1093/nop/npae017)
Supplement: npae017_suppl_Supplementary_Material [file npae017_suppl_supplementary_material.docx]

**Global health status and fatigue score in IDH-mutant diffuse glioma grades 2 and 3 – A longitudinal population-based study from surgery to 12-month follow-up.**

**1. Baseline characteristic, treatments, and all patients included at baseline.**

Supplementary table with patient characteristics, treatments, and clinical outcomes (N=56).

| **Variable** | **Study sample** |
| --- | --- |
| Age at surgery, mean (SD) | 44.3 (13.9) |
| Female, No (%) | 26 (46.4) |
| KPS at admission, median (Q1, Q3) | 90 (80-90) |
| Incidental, No (%)  History of seizures at admission, No (%) | 9 (16.1)  37 (66.1) |
| Neurological deficit at admission, No (%) |  |
| Motor | 3 (5.4) |
| Cognitive | 9 (16.1) |
| Visual | 3 (5.4) |
| Language | 6 (10.7) |
| Any neurological deficit at admission, No (%) | 18 (32.3) |
| Tumor location, No (%) |  |
| Mainly frontal | 32 (57.1) |
| Mainly temporal | 9 (16.1) |
| Other | 15 (26.8) |
| Choice of neurosurgical intervention, No (%) |  |
| Tumor resection | 51 (91.1) |
| WHO 2021 classification, No (%) |  |
| Oligodendroglioma, grade 2 | 14 (25.0) |
| Astrocytoma, grade 2 | 21 (37.5) |
| Oligodendroglioma, grade 3 | 14 (25.0) |
| Astrocytoma, grade 3 | 7 (12.5) |
| Treatment before 12-month follow-up, No (%) |  |
| None | 9 (16.1) |
| Chemotherapy only ^a^ | 7 (12.5) |
| Radiochemotherapy ^a, b^ | 40 (71.4) |
| Other relevant treatments and interventions, No (%) |  |
| Antiepileptic drug use at baseline | 37 (66.1) |
| Antiepileptic drug use at 12-month follow-up, (N=51) | 36 (64.3) |
| Physical rehabilitation, (N=55) | 10 (17.9) |
| Post-operative seizures ^c^, No (%) | 7 (12.5) |
| New or worsened neurological deficit ^d^, No (%) |  |
| Motor | 13 (23.2) |
| Cognitive | 8 (14.3) |
| Visual | 2 (3.6) |
| Language | 12 (21.4) |
| Any new or worsened neurological deficit, No (%) | 25 (44.6) |
| Any permanent, new or worsened, neurological deficit, No (%) | 14 (25.0) |

^a^ Either temozolomide, lomustine or procarbazine hydrochloride, lomustine and vincristine (PCV).

^b^ Either concomitant or adjuvant.

^c^ From surgery up to the 3-month follow-up. New or worsened.

^d^ From surgery up to the 3-month follow-up. Transient or permanent.

**2. Baseline characteristic, treatments, and outcomes by tumor type.**

Supplementary table comparing patient characteristics, treatments, and clinical outcomes between patients with oligodendroglioma and patients with astrocytoma.

| **Variable** | **Oligodendroglioma**  **(n=28)** | **Astrocytoma**  **(n=28)** | **p-value** ^a^ |
| --- | --- | --- | --- |
| Age at surgery, mean (SD) | 50.5 (13.0) | 38.2 (12.1) | <0.01 |
| Female, No (%) | 14 (50) | 12 (42.9) | 0.79 |
| KPS <80 at admission, No (%) | 4 (14.3) | 2 (7.1) | 0.67 |
| Incidental | 3 (10.7) | 6 (21.4) | 0.47 |
| History of seizures, No (%) | 19 (67.9) | 18 (64.3) | 1.00 |
| Neurological deficit at admission, No (%) |  |  |  |
| Motor | 2 (7.1) | 1 (3.6) | 1.00 |
| Cognitive | 5 (17.9) | 4 (14.3) | 1.00 |
| Visual | 1 (3.6) | 2 (7.1) | 1.00 |
| Language | 3 (10.7) | 3 (10.7) | 1.00 |
| Any neurological deficit at admission, No (%) |  |  |  |
| Choice of neurosurgical intervention, No (%) |  |  |  |
| Tumor resection | 27 (96.4) | 24 (85.4) | 0.35 |
| WHO 2021 grade, No (%) |  |  |  |
| Grade 2 | 14 (50) | 21 (75) | 0.10 |
| Treatment before 12-month follow-up, No (%) |  |  |  |
| None | 5 (17.9) | 4 (14.4) | 1.00 |
| Chemotherapy only ^b^ | 5 (17.9) | 2 (7.1) | 0.42 |
| Radiochemotherapy ^b,c^ | 18 (64.3) | 22 (78.6) | 0.38 |
| Other relevant interventions//treatments, No (%) |  |  |  |
| AED at baseline | 21 (75) | 16 (57.1) | 0.26 |
| AED at 1 year | 21 (75) | 15 (53.6) | 0.36 |
| Physical rehabilitation | 5 (17.9) | 5 (17.9) | 1.00 |
| Post-operative seizures ^d^, No (%) | 2 (7.1) | 5 (17.9) | 0.42 |
| New or worsened neurological deficit ^e^, No (%) |  |  |  |
| Motor | 8 (28.6) | 5 (17.9) | 0.53 |
| Cognitive | 5 (17.9) | 3 (10.7) | 0.71 |
| Visual | 1 (3.6) | 1 (3.6) | 1.00 |
| Language | 6 (21.4) | 6 (21.4) | 1.00 |
| Any new or worsened neurological deficit, No (%) | 15 (53.6) | 10 (35.7) | 0.28 |
| Any permanent new or worsened neurological deficit, No (%) | 8 (28.6) | 6 (21.4) | 0.76 |

^a^ Independent t-test or Fisher-Freeman-Halton exact test.

^b^ Either temozolomide, lomustine, or procarbazine hydrochloride, lomustine and vincristine (PCV).

^c^ Either concomitant or adjuvant.

^d^ From surgery up to the 3-month follow-up. New or worsened.

^e^ From surgery up to the 3-month follow-up. Transient or permanent.

**3. Baseline, 3-month and 12-month follow up including all scales and items of the QLQ-C30 and QLQ-BN20 at group level for all patients.**

| **QLQ-C30 and QLQ-BN20**  **scales and single items** | **Baseline (n=56)**  mean (SD) | **3-month (n=50)**  mean (SD) | **12-month (n=51)**  mean (SD) |
| --- | --- | --- | --- |
| Global health status **(*)** | 58.3 (22.8) | 63.0 (21.4) | 61.6 (24.5) |
|  |  |  |  |
| Physical functioning **(*)** | 87.4 (17.6) | 83.3 (20.8) | 83.7 (20.2) |
| Role functioning | 62.8 (35.8) | 60.7 (35.9) | 66.7 (35.6) |
| Emotional functioning | 61.0 (26.6) | 69.8 (26.2) | 72.6 (26.7) |
| Cognitive functioning **(*)** | 75.0 (24.4) | 73.7 (26.1) | 76.0 (24.6) |
| Social functioning | 72.0 (30.0) | 73.0 (29.1) | 74.2 (30.2) |
|  |  |  |  |
| Pain | 19.0 (23.9) | 16.7 (22.8) | 15.4 (23.3) |
| Fatigue **(*)** | 37.3 (29.6) | 41.1 (26.4) | 36.7 (28.4) |
| Nausea/vomiting | 9.5 (18.8) | 7.7 (12.2) | 9.5 (16.4) |
|  |  |  |  |
| Dyspnea | 21.4 (27.3) | 25.3 (29.8) | 27.5 (30.3) |
| Insomnia | 33.3 (31.8) | 22.7 (26.5) | 24.8 (29.0) |
| Appetite loss | 16.1 (30.0) | 12.7 (23.2) | 16.3 (26.1) |
| Constipation | 14.3 (26.9) | 8.7 (21.1) | 11.1 (22.8) |
| Diarrhea | 12.5 (22.5) | 8.7 (18.8) | 8.5 (20.9) |
| Financial problems | 16.7 (27.7) | 23.3 (31.0) | 19.0 (32.8) |
|  |  |  |  |
| Future uncertainty | 39.6 (24.2) | 31.2 (24.6) | 22.1 (21.7) |
| Vision disorder **(*)** | 11.9 (19.9) | 9.6 (17.0) | 9.2 (14.9) |
| Motor dysfunction **(*)** | 10.9 (18.8) | 13.6 (22.4) | 13.7 (24.4) |
| Communication deficit **(*)** | 14.9 (18.7) | 16.9 (19.9) | 19.0 (23.8) |
|  |  |  |  |
| Headaches | 31.0 (32.3) | 18.7 (27.9) | 17.7 (24.4) |
| Seizures **(*)** | 6.6 (14.8) | 3.3 (12.1) | 5.2 (16.8) |
| Drowsiness | 34.5 (33.0) | 33.3 (29.4) | 36.6 (29.3) |
| Itchy skin | 5.4 (15.3) | 14.0 (24.4) | 13.1 (26.7) |
| Weakness of legs | 19.7 (22.1) | 11.3 (19.8) | 7.8 (19.5) |
| Bladder control | 4.2 (11.1) | 4.7 (16.5) | 6.6 (17.7) |
| Hair loss | 3.6 (15.2) | 26.7 (34.3) | 8.5 (19.8) |

^(*)^ Selected scales and items highlighted in the article.

**4. Individual change in QLQ-C30 and QLQ-BN20 selected scales and items at 12-month follow-up between patients diagnosed with oligodendroglioma and patients diagnosed with astrocytoma.**

| **Change in QLQ-C30 and QLQ-BN20 at 12-month follow-up**^1^ | **Oligodendroglioma (n=26)**  mean (SD) | **Astrocytoma (n=25)**  mean (SD) | **p-value**^1^ |
| --- | --- | --- | --- |
| Status and functioning scores^2^: |  |  |  |
| Global health status | 0.6 (28.2) | -10.0 (20.0) | 0.13 |
| Physical functioning | 9.0 (11.6) | -0.8 (13.1) | 0.01 |
| Cognitive functioning | -3.8 (24.2) | 1.0 (23.9) | 0.47 |
|  |  |  |  |
| Dysfunctions, deficits, and symptoms^3^: |  |  |  |
| Motor dysfunction | -7.3 (23.7) | -1.3 (12.1) | 0.27 |
| Vision disorder | 2.9 (19.5) | 4.4 (13.2) | 0.76 |
| Communication deficit | -5.6 (19.7) | -1.3 (16.5) | 0.41 |
| Fatigue | -1.9 (25.1) | 5.3 (25.9) | 0.31 |
| Seizures | 3.9 (19.6) | 0.0 (21.5) | 0.51 |

^1^ Independent t-test.

^2^ In status and functioning scores negative change indicates improvement.

^3^ In dysfunctions, deficits and symptoms scores positive change indicates improvement.

**5. Significant Spearman rank order partial correlations including** **all patient characteristics and individual change in QLQ-C30 and QLQ-BN20 selected scales and items at the 12-month follow-up.**

|  |  | Spearman correlation coefficient (r) | 95% confidence interval (CI) | p-value |
| --- | --- | --- | --- | --- |
| Cognitive deficit at admission | Visual deficit at admission | -0.8 | (-0.9 to -0.5) | <0.001 |
| Cognitive deficit at admission | Language deficit at admission | -0.9 | (-1 to -0.8) | <0.001 |
| Cognitive deficit at admission | Any neurological deficit at admission | 0.9 | (0.8 to 1) | <0.001 |
| Language deficit at admission | Visual deficit at admission | -0.8 | (-0.9 to -0.55) | <0.001 |
| Language deficit at admission | Any neurological deficit at admission | 0.9 | (0.7 to 1) | <0.001 |
| Visual deficit at admission | Any neurological deficit at admission | 0.9 | (0.6 to 0.9) | <0.001 |
| New or worsened motor deficit at 3-month | New or worsened visual deficit at 3-month | -0.7 | (-0.9 to -0.4) | <0.001 |
| New or worsened language deficit at 3-month | Any new or worsened neurological deficit at 3-month | 0.8 | (0.5 to 0.9) | <0.001 |
| No postoperative oncological treatment before 12-month follow-up | Chemotherapy only oncological treatment before 12-month follow-up | 0.8 | (0.5 to 0.9) | <0.001 |

**6. Statistically significant single predictors in univariable linear regression with change in the fatigue score at the 12-month follow-up as response variable (N=51).**

|  | R2 | df (regression, residual) | F | p-value |
| --- | --- | --- | --- | --- |
| AED use at 12-month follow-up: the overall regression was statistically significant | 0.11 | (1, 44) | 5.41 | 0.025 |
| Chemotherapy only before 12-month follow-up: the overall regression was statistically significant | 0.09 | (1, 49) | 4.77 | 0.034 |

**7. Global health status and fatigue scores at baseline, 3 and 12-month follow-up in patients grouped by unfavorable change beyond MCID in global health status and fatigue score.**

|  | **Global health status at baseline** (n=51).  **Mean (SD)** | **Global health status at 3-month follow-up** (n=49).  **Mean (SD)** | **Global health status at 12-month follow-up** (n=51).  **Mean (SD)** |
| --- | --- | --- | --- |
| Patients with unfavorable change in global health status, n=12 | 72.9 (18.8) | 58.3 (20.1) | 41.7 (19.8) |
| -All other patients | 52.1 (22.3) | 64.2 (22.0) | 67.7 (22.6) |
| Patients with unfavorable change in fatigue scores, n=17 | 65.2 (25.0) | 60.3 (22.7) | 51.0 (25.5) |
| -All other patients | 52.9 (21.4) | 64.1 (21.1) | 66.9 (22.5) |

|  | **Fatigue scores at baseline** (n=51).  **Mean (SD)** | **Fatigue scores at 3-month follow-up** (n=49).  **Mean (SD)** | **Fatigue scores at 12-month follow-up** (n=51).  **Mean (SD)** |
| --- | --- | --- | --- |
| Patients with unfavorable change in global health status, n=12 | 32.4 (27.4) | 49.1 (21.4) | 54.2 (21.5) |
| -All other patients | 40.2 (29.5) | 39.0 (27.8) | 31.3 (28.4) |
| Patients with unfavorable change in fatigue scores, n=17 | 26.1 (26.0) | 46.4 (20.5) | 53.3 (24.3) |
| -All other patients | 44.4 (28.7) | 38.9 (29.2) | 28.4 (27.0) |
